# Unlocking the potential of *in silico* chemical safety assessment – A report on a cross-sector symposium on current opportunities and future challenges

**DOI:** 10.1016/j.comtox.2018.12.006

**Published:** 2019-05

**Authors:** Mark T.D. Cronin, Judith C. Madden, Chihae Yang, Andrew P. Worth

**Affiliations:** a Liverpool John Moores University, School of Pharmacy and Biomolecular Sciences, Byrom Street, Liverpool L3 3AF, United Kingdom; b Molecular Networks GmbH, Neumeyerstraße 28, 90411 Nürnberg, Germany; c European Commission, Joint Research Centre (JRC), Ispra, Italy

**Keywords:** Read-across, Quantitative structure-activity relationships (QSARs), Computational toxicology, Exposure, Informatics

## Abstract

*In silico* chemical safety assessment can support the evaluation of hazard and risk following potential exposure to a substance. A symposium identified a number of opportunities and challenges to implement *in silico* methods, such as quantitative structure-activity relationships (QSARs) and read-across, to assess the potential harm of a substance in a variety of exposure scenarios, e.g. pharmaceuticals, personal care products, and industrial chemicals. To initiate the process of *in silico* safety assessment, clear and unambiguous problem formulation is required to provide the context for these methods. These approaches must be built on data of defined quality, while acknowledging the possibility of novel data resources tapping into on-going progress with data sharing. Models need to be developed that cover appropriate toxicity and kinetic endpoints, and that are documented appropriately with defined uncertainties. The application and implementation of *in silico* models in chemical safety requires a flexible technological framework that enables the integration of multiple strands of data and evidence. The findings of the symposium allowed for the identification of priorities to progress *in silico* chemical safety assessment towards the animal-free assessment of chemicals.

## 1 Introduction

**Fig. 1:**
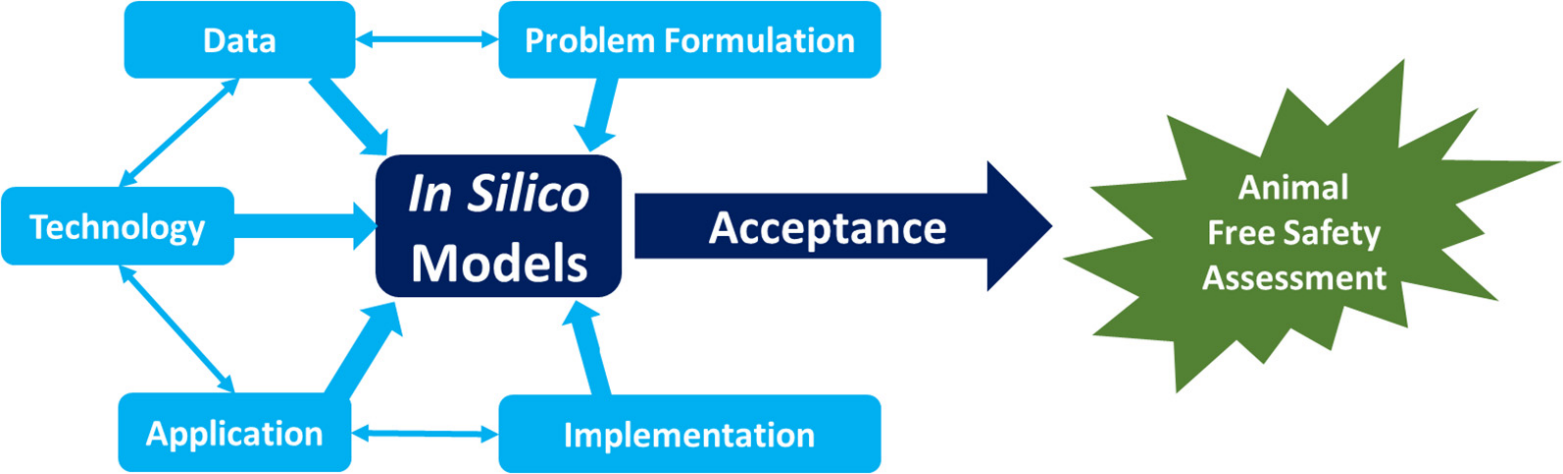
The main areas of opportunities and challenges identified for *in silico* models to support animal-free safety assessment of chemicals.

*In silico* chemical safety assessment aims to place the evaluation of the risks of chemical exposure on a much broader and profound knowledge-base. It has at its heart the need for more robust, reproducible, translatable, cheaper, rapid and ethically acceptable assessment of chemicals. This topic was the main focus of a forward-looking symposium held on 11 October 2018 at the Friedrich-Alexander-University, Erlangen, Germany. The purpose of the symposium was to reflect on perspectives, opportunities and challenges in chemical safety and to find future directions. The key drivers for the symposium were to investigate the use of *in silico* approaches in safety assessment with a focus on quantitative structure-activity relationships (QSARs) and read-across. Key stakeholders and contributors from the pharmaceutical, personal care products and chemical industries, as well as from regulatory agencies, were invited to make presentations. In particular, the symposium enabled cross-industry discussions and an opportunity to develop and share a vision for the next steps in this field. This report summarises the main findings that came from a series of expert presentations and discussions; it is based around a number of themes that arose concerning the on-going development of models that will allow for animal-free chemical assessment. The main themes and their inter-relationships are summarised in Fig. 1, with a clear emphasis on cross-sector appreciation of the issues and means to resolve them.

## 2 Problem formulation

*In silico* techniques are used in a wide variety of scenarios within and between industries including, but not limited to, screening, prioritisation, classification and labelling, risk assessment, and product development [1]. While there is no “one size fits all” approach for *in silico* modelling, and approaches must be tailored to the specific use case and context, there are a number of common themes. One of the key themes is the motivation of industries to adopt the “fail-fast, fail-cheap” philosophy to screen out potentially hazardous molecules early in the development process [2]. However, other specific uses of *in silico* methods abound. For instance, within the pharmaceutical industry, knowledge-based systems and QSARs are used to predict mutagenicity of impurities as part of the ICH Harmonised Guideline M7 scheme [3], [4]. At the other end of the spectrum, cosmetics industries foresee the use of *in silico* techniques as part of an *ab initio* approach to assess the overall impact of a chemical. The assessment typically includes information on mechanisms of action, exposure, and use case scenarios, as well as the more traditional and accepted use for toxicity prediction [5]. Increasingly, attention is also being turned to consideration of formulated products, mixtures and natural products in addition to the “traditional” application to single substances. A number of common drivers are apparent for the use of *in silico* approaches across the sectors relating in part to the replacement of animal use and to more human-relevant information offering improved scientific practice. There is a clear requirement for *in silico* modelling to cross geographical boundaries and industrial sectors, to learn from each other and enable knowledge to be applied effectively.

Overall, while there is extensive development of new *in silico* methods, there is an overriding need to ensure these are fit for purpose. This implies close collaboration between chemists, toxicologists, informaticians and risk assessors to develop, maintain and utilise appropriate models. Not only must the different disciplines come together, but also those scientists from industry, academia and regulatory agencies must recognise the commonalities. The challenge is to respond to the growing need for adaptable, flexible and even bespoke computational workflows that meet the demands of industry and regulators, by exploiting the emerging methodologies of 21st Century toxicology and risk assessment.

## 3 Data

Toxicological data and information are crucial to *in silico* safety assessment not only in terms of availability, but also their abundance and quality. The current status is an increasing number of data relating to the adverse effects of chemicals which range from the results of high content analyses to historical toxicity data across a number of publicly and commercially available databases. There is also a wealth of (potentially high quality) toxicological data buried in the archives of business, and pharmaceutical companies in particular, which would offer great opportunities, if exploited. This has brought forward the concept of data sharing to enable access to otherwise untapped resources. It is known that data sharing requires overcoming the apprehension of (pharmaceutical) companies about the release of sensitive, proprietary, preclinical data. Two international initiatives, the eTOX and the eTRANSAFE projects, have shown how sharing data (previously considered to be commercially sensitive) could be achieved, on a with-cost basis, with the former project showing demonstrable success and promise for the future [6], [7], [8], [9]. The development of these databases from in-house data complement other activities, such as the freely available COSMOS DB [10], which have focussed on sharing data for non-pharmaceutical compounds, e.g. cosmetics ingredients and fragrances. These projects have helped identify and resolve a number of problems; for instance, integration of data from different sources requires the development and implementation of ontologies and other standards – eTOX being an example where effort was made to create standardised ontologies [11]. In this respect, the CDISC SEND format is a standard that will facilitate the sharing of preclinical legacy data and, consequently, assist the development of more reliable (in terms of not only predictive performance, but also applicability and translatability) *in silico* predictive systems.

Another recognised issue with public and private data resources alike is ensuring and understanding the quality of the data. Understanding data quality involves not only accurate recording and translation of data, i.e. ensuring what is stored in the database is accurate through appropriate annotation and curation, but also evaluating the value and relevance of the data according to the context of use [12]. All efforts to create reliable and usable databases require expert judgement of quality and data checking through defined procedures. Expert judgement also assists in the evaluation of data, as demonstrated by their utility in models, and hence checking and updating of data resources. Appreciation of data quality will enable higher confidence in models and understanding of their limitations [13].

The opportunity for the future use of toxicological data is to encourage and embrace data sharing, recognising all the practical and legal problems particularly around standardisation and implementation. Techniques such as the use of honest brokers and masking of chemical identity will enable full use of data whilst ensuring confidentiality. A snowball effect is often seen, when one company releases data, others are encouraged to do so. The advantages (chemical diversity, reliability, quality, granularity of information) clearly outweigh the problems and costs.

## 4 Models

The modelling of toxicity and the use of the subsequent models within an appropriate modelling framework is at the heart of the *in silico* safety paradigm. There is an increasing variety of models to predict toxicity varying in their application from the use of structural alerts to artificial intelligence and deep learning algorithms. This is accompanied by a burgeoning realisation of the need to change focus from single standalone models to their role within frameworks that can integrate and interpret multiple strands of data and information. Of the models available there is a currently a particular focus on read-across in terms of identifying analogues and providing supporting data to make better use of existing experimental toxicity data [14], [15]. This is seen as a powerful tool for data gap filling, with successes in areas such as the completion of dossiers for REACH, although with many questions still left unanswered, such as those relating to acceptance and the translatability to humans and, for environmental risk assessment, a broader spectrum of species. There is increasing coverage of structural alerts for many endpoints that have previously proven difficult to model [16], [17] with further work on-going.

Many exciting and novel applications are seen in modelling and predicting “challenging” properties and endpoints. For instance, one of the most difficult aspects to predict is the formation of metabolites. To address this, a new programme MetScore predicts Sites of Metabolism (SoM) for Phase I and Phase II metabolic transformations. The software is based on specifically developed radial atomic reactivity and steric descriptors. This enables the optimisation of compounds in drug discovery projects with regard to metabolic stability [18]. Other work is developing better fingerprints for chemical and biological similarity. For instance, new Vectorised Phenotype Descriptors have been developed from High Content Screening FingerPrints (HCSFP) on the basis of image analysis from cellular high content screening [19]. The HCSFPs can be used to cluster compounds, providing different information to chemical similarity alone. This work enables the linkage of phenotype to Mode of Action, with the assumption that similarity in the HCSFP descriptors is related to activity. This method allows the user to interrogate the original information, in terms of the image analyses, to resolve any potential queries relating to activity.

In addition to developing novel modelling tools, new schemes and techniques for modelling are required for safety assessment in particular areas. Modelling approaches can be applied to mixtures (in the broadest sense including co-exposures, natural products etc). The EU EuroMix Project is developing strategies to implement *in silico* approaches for Cumulative Assessment Groups (used in the EU assessment of pesticides) as part of this initiative (https://www.euromixproject.eu/). Different approaches can be applied to mixtures from simplistic Thresholds of Toxicological Concern (TTC) for individual components to establishing similarity in mode of toxic action [20]. *In silico* approaches can be applied at various levels to model mixture toxicity from phenotype to organ effects to mode and mechanism of action. QSAR and molecular docking approaches have been applied to evaluate the similarity of compounds that elicit the same mechanism of liver toxicity as well as other effects such as reproductive and developmental toxicity. Modelling of mixture toxicity will require the unravelling of mechanisms, interactions and toxicity data as well as acceptance of new strategies and, inevitably, new criteria for acceptance.

It is, of course, the model that is central to all other issues considered in this report: while data and technologies support the effort, the model itself holds the key for application and implementation. The opportunities and challenges for models are intrinsically linked, with the need for proper problem formulation being required to inform model implementation and acceptance, and *vice versa*. It is well known that despite a half century of progress in QSAR, there are still many toxicological endpoints that are difficult to predict, e.g. chronic toxicity, developmental and reproductive toxicity [21]. It is even arguable that some of these chronic effects, involving long periods from insult-to-injury, are not even predictable, except in probabilistic terms. In addition, there is chemical diversity both within, but more significantly between, industrial sectors, which raises the challenge of domain-specific or more general models. The opportunity is to provide fit-for-purpose and chemistry relevant predictive systems based on reliable, quality assured toxicological data. New systems will need to integrate different types of data to address issues of similarity, from both the chemical and biological perspective. Likewise, there is a need to consider dissimilarity both in terms of read-across analogues and the use of model applicability domains. At the moment, there are many key questions but none more important than checking whether a compound is within the applicability domain of a model, and knowing how to deal with a compound outside of the domain. This is also linked to the acceptance of a prediction, i.e. when does a model cease to be sufficiently predictive, or when is there too much uncertainty surrounding the prediction? Opportunities for addressing such questions arise from approaches that quantify the uncertainty of prediction (based on performance statistics), using techniques such as Dempster-Shafer Theory to combine uncertainties within a weight-of-evidence modelling approach [22].

## 5 Technology

The models and data repositories for *in silico* chemical safety assessment require appropriate tools for their implementation. New informatics structures have enabled the linking of data and predictive resources in tools such as ChemTunes and ToxGPS [23]. From the description of molecular properties to adverse outcomes, there are various sophisticated means of capturing relevant information. The European Food Safety Authority (EFSA) has taken the approach of developing technologies and tools that will allow for the application of (Q)SAR and read-across as well as linkage to relevant databases e.g. EFSA OpenFoodTox [24].

The new technologies also need to incorporate progress in the understanding and modelling of the biological mechanisms from a systems biology perspective. This will ultimately facilitate the discovery of new targets for pharmacological interventions, and enable the design and development of new drugs in a way that reduces the risk of the undesired side-effects.

The opportunity is to draw together the various strands of *in silico* chemical safety assessment such as chemistry, toxicology and informatics into an integrated “data science” platform that allows for the formulation of useable knowledge derived from the information and data within the databases. Such a platform would enable decisions to be informed by the *in silico* evidence. Future *in silico* chemical safety assessment tools need to be flexible and future-proof, allowing a user to move seamlessly between different levels of the Data-Information-Knowledge pyramid.

## 6 Application and implementation

The application of models and tools in *in silico* chemical safety assessment is intrinsically linked to the problem formulation [25]. Models and tools must be adaptable in a business environment where markets are global, but most regulations are regional. The implementation of *in silico* chemical safety assessment requires the use of expert judgement. To obtain a satisfactory outcome, i.e. acceptance of a prediction, guidance is required – for regulatory decisions this would normally be derived from the relevant regulatory body (e.g. guidance issued by the European Chemicals Agency (ECHA), EFSA or the European Medicines Agency (EMA)). Guidance needs clear practical instructions to ensure reproducibility; this is reinforced by the necessity of clear and transparent documentation to minimise subjectivity and allow for reproducibility in conducting and reporting *in silico* toxicology. In addition to undertaking the predictions, there is a need to understand the uncertainties associated with predictions and how the uncertainties can be defined and usefully applied. For instance, there is still considerable, and often unknown, uncertainty in the source data. Case studies demonstrating the practical implementation of the guidance for *in silico* chemical safety assessment are also helpful as an educational resource. There is also a recognised need for capacity building of trained users within a multidisciplinary international community [26].

Models are no longer applied in isolation to determine chemical safety; there has been a growing global trend towards the development and use of multiple strands of information within Integrated Approaches to Testing and Assessment (IATA) for safety assessment [5], [27], [28]. Nearly all IATA involve the use of existing data, (Q)SAR predictions and/or read-across that are amenable to being integrated into computational workflows. In addition, there is a growing trend towards weight-of-evidence approaches that combine different types of predictions, e.g. for ICH M7, to reach an overall conclusion which inevitably involves some degree of expert judgement.

To enable safety assessment to be undertaken, there is a clear need to include sufficient information about exposure, including internal exposure. Much of this will rely on data-driven expert judgement, supported where possible by QSARs – although it is acknowledged that reliable QSARs are lacking for many ADME endpoints. Exposure models, such as Physiologically Based Kinetic (PBK) models [29], will be strengthened by including experimental data; for instance there is great value in including *in vitro* data (e.g. from high throughput screening) to strengthen models for ADME properties [30]. Different exposure models are required by the different industrial sectors, e.g. while the cosmetics industry is primarily concerned with the dermal and inhalation routes, oral absorption is more important for the pharmaceutical and food industries. The TTC approach is seen as a simple and practical means of safety assessment based on information on chemical structure and exposure alone [31] ; it may also assist in the evaluation of mixtures of synthetic and natural products. There is an increasing need to consider internal exposures to chemicals, again through the modelling of the distribution of chemicals. Such modelling may allow for the derivation, in the future, of reliable “internal TTC” (iTTC) values. Since assessments of exposure and knowledge of (toxicologically relevant) points of departure are at the heart of modern safety assessment, providing the modelling support to make the assessments, and hence decisions, will be particularly important in areas such as cosmetics and personal products.

From a business perspective, there must be realism with regard to software. While transparency in modelling and frameworks is preferred, this must be reconciled with the issues of Intellectual Property Rights (IPR) and commercial interests to maintain a product. Transparency in the definition of the models within commercial systems and how decisions are reached is required. This is a critical aspect to avoid “black-box” systems where a prediction is made but cannot be justified or verified. There is a balance, however, to maintain the sustainability and commercial viability in the development of models and software which incorporates appropriate transparency while protecting the IPR of the developer. While there are considerable advantages in using freely available software and databases, it must be remembered that there is a cost associated with all *in silico* resources. Sustainable business models are essential across the industry that allow for free access, where appropriate, for instance for training, education and increasing awareness. However, to ensure the future legacy and viability of models and resources, as well as appropriate maintenance, support and security, there must be realism and the acceptance of cost and need for funding on a commercial basis. Such funding will enable other aspects of implementation, such as functionality (extensibility and sharing) and versioning, to be maintained and developed.

The opportunity is for the application and implementation of safety assessment methodologies in computational frameworks, based on a sustainable business model. The tools need to be flexible to account for needs at the business and regulatory levels. Guidance, protocols and training are needed to develop understanding, transparency and consistency in the application of methods, and to increase the number of trained users.

## 7 Acceptance

*In silico* chemical safety assessment must be fit for purpose, which can be interpreted as being acceptable for the specific scientific question and use context. Acceptability is the willingness of the end-user (e.g. regulatory assessor) to trust the model and its predictions, and thus does not represent the sole belief of the model developer. The type and level of acceptance required will influence how modelling is undertaken and reported. Regulatory toxicology, to a large extent, is based on a culture of “validation” – especially when it comes to acceptance of alternative approaches over traditional animal tests. While the OECD Principles for Validation of QSARs are intended to support the regulatory application of models, it can be difficult to translate them into an “acceptable prediction”, or what in REACH guidance is termed an “adequate (Q)SAR Result” [32]. Much has been made of acceptance of read-across predictions for the REACH legislation, but this is on a case-by-case basis and cannot be guaranteed *a priori*. Acceptance can however be influenced by demonstrating the confidence associated with a prediction (e.g. through definition of uncertainties) as well as careful and thorough documentation providing access to the underlying data [33]. It is inevitable that high confidence will be required in some instances, e.g. risk assessment, to ensure acceptability. It is recognised that, for good reasons, the regulatory frameworks that are the result of the political and legislative process are designed to give a degree of certainty and consistency to all stakeholders and therefore may lack the mechanisms to adapt quickly to the progress made in *in silico* science. This has led to frustrations on all sides and a realisation that there is a need to optimise the process for acceptance and use of *in silico* methods.

The opportunity for *in silico* chemical safety assessment, as a community of developers, users and evaluators, is to provide assessments of the confidence that may be associated with predictions in order to determine their utility. Once the required level of confidence is established for a given application and context, acceptance for predictions can be sought and case-dependent, evidence-based solutions provided.

## 8 Conclusions

**Fig. 2:**
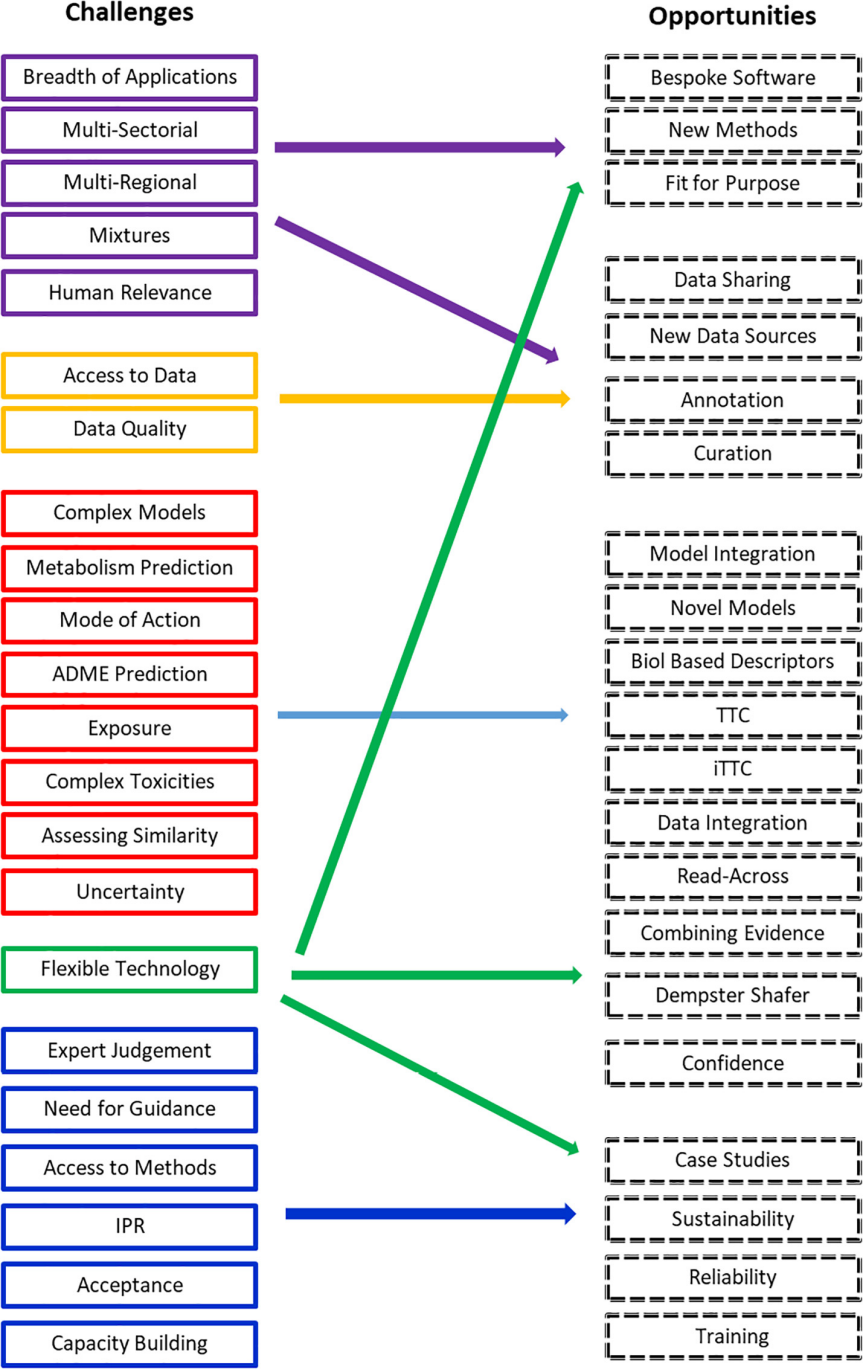
The main challenges for *in silico* chemical safety assessment and their interrelationship with the opportunitiesidentified during the symposium. For the challenges, the boxes are colour coded according to five main themes: purple – problem formulation; gold – data: red – models; green – technology; blue – implementation, application and acceptance. The opportunities cross all the main issues identified.

*In silico* chemical safety assessment is at the confluence of informatics, chemistry, biology, mechanistic toxicology and regulatory science. It provides a platform to make evidence-informed decisions which are defensible and fit-for-purpose. The challenges and related opportunities identified within the symposium are summarised in Fig. 2. While this is not a comprehensive overview, it does provide a snapshot of the current status of *in silico* chemical safety assessment. An obvious feature is the challenge to provide a flexible technologies which will form the basis of the new modelling approaches and allow them to be utilised appropriately and successfully.

The overriding goal of *in silico* chemical safety assessment is the acceptance of a prediction for a particular purpose, while the challenges to achieve that goal are many and varied. Key to acceptance is the ability to provide a well-documented body of evidence meeting a defined level of confidence and to ensure proper and appropriate use and avoid misuse for business, political or other purposes. However, a key remaining challenge is how to identify, quantify and consistently communicate uncertainties (especially related to New Approach Methodologies) – a process which is often subjective and usually performed on a case-by-case basis. Other challenges faced in making *in silico* scientific methods acceptable are partly technical as well as political, socio-economic and cultural [34]. Within the challenges, there are considerable opportunities to extend the current paradigm in toxicology and risk assessment and bring together many disparate disciplines to strive towards animal-free, and target species relevant, assessments that are broadly applicable across the globe.
